# Self-Improvising Memory: A Perspective on Memories as Agential, Dynamically Reinterpreting Cognitive Glue

**DOI:** 10.3390/e26060481

**Published:** 2024-05-31

**Authors:** Michael Levin

**Affiliations:** Department of Biology, Allen Discovery Center, Tufts University, 200 Boston Avenue, Suite 4600, Medford, MA 02155-4243, USA; michael.levin@tufts.edu; Tel.: +1-617-627-6161

**Keywords:** basal cognition, diverse intelligence, memory, learning, morphogenesis

## Abstract

Many studies on memory emphasize the material substrate and mechanisms by which data can be stored and reliably read out. Here, I focus on complementary aspects: the need for agents to dynamically reinterpret and modify memories to suit their ever-changing selves and environment. Using examples from developmental biology, evolution, and synthetic bioengineering, in addition to neuroscience, I propose that a perspective on memory as preserving salience, not fidelity, is applicable to many phenomena on scales from cells to societies. Continuous commitment to creative, adaptive confabulation, from the molecular to the behavioral levels, is the answer to the persistence paradox as it applies to individuals and whole lineages. I also speculate that a substrate-independent, processual view of life and mind suggests that memories, as patterns in the excitable medium of cognitive systems, could be seen as active agents in the sense-making process. I explore a view of life as a diverse set of embodied perspectives—nested agents who interpret each other’s and their own past messages and actions as best as they can (polycomputation). This synthesis suggests unifying symmetries across scales and disciplines, which is of relevance to research programs in Diverse Intelligence and the engineering of novel embodied minds.

## 1. Introduction and Overview

*“To live is to be other. It’s not even possible to feel, if one feels today what he felt yesterday. To feel today what one felt yesterday is not to feel—it’s to remember today what was felt yesterday, to be today’s living corpse of what yesterday was lived and lost. To erase everything from the slate from one day to the next, to be new with each new morning, in a perpetual revival of our emotional virginity—this, and only this, is worth being or having, to be or have what we imperfectly are.”*

Fernando Pessoa

 

There is a paradox which points out that if a species fails to change, it will die out, but if it changes, it likewise ceases to exist. The same issue faces all of us: if we do not change, learning and growth is impossible. If we do change, does not the current Self cease to exist, in an important sense? This profound puzzle, which rests on pure logic—not contingent facts of implementation or origin—highlights the deep symmetry of existential concerns for agents existing at all scales, from subcellular organelles to evolutionary lineages^1^. Thus, it is also highly relevant to issues facing the engineering of novel intelligences and fields from active matter research to AI and Artificial Life.

A solution to this problem has been suggested in the West as Process Philosophy [10,11,12], and in the East as Buddhist and Indic conceptions of the no-Self or the non-duality of the Self vs. the world [13]. The idea is to conceive of the Self^2^ as a process, not a thing, and to consider snapshot Selves^3^ as low-dimensional projections of the deeper reality that both exists and constantly changes. Here, I discuss how evolution has beaten us to this solution, and how it is implemented across spatiotemporal scales. Biology went all in, committing to the fact that everything will change in ways that cannot be anticipated, and thus to the idea that defending static Selves is futile. When it comes to information, biology commits to optimizing salience and meaning [15,16,17], not fidelity, via high-level, agent-based interpretation—the gestalt, not the low-level details. Crucially, this is not just about life forms preparing for challenging external environments—it is even more about the inevitable changes of our *internal* parts, which are subject to mutation, aging, cancer, and hacking by other biota. More broadly, it is about the passage of time.

The essential unreliability of the biological substrate was a key driver of an architecture that is responsible for numerous fascinating capacities of the minds and bodies of life forms. Here, I discuss our nature as a hierarchy of *essentially epistemically vulnerable*^4^ agents, who must interpret the actions of our own parts and information structures despite their being prone to error, decay, and autonomous behaviors. I argue that this is not a limitation of living things but rather it is the origin of life’s most unique aspects, and of intelligence specifically. I provisionally call *mnemonic improvisation* the dynamic ability to re-write and remap information (e.g., memories) onto new media and new contexts, which occurs at many scales (behavioral, genetic, and physiological memories). Thus, I propose that the ability to modify and interpret non-local memories is one of the kinds of cognitive glue that reifies Selves (at all scales) [23,24,25].

One specific example concerns the maintenance of training-induced memories across metamorphosis, such as that from caterpillar to butterfly [26,27]. I focus not on the puzzle of how memories can remain despite drastic refactoring of the brain tissue, but on the deeper puzzle of how memory engrams can be interpreted and used adaptively by a completely different body, in the context of which the details of the original memory would be useless. I focus on a bowtie architecture (prominent across biological signaling and in autoencoders in machine learning) as an example of how compression during learning and generalization must involve creative reinterpretation upon recall [28]. This schema can be applied widely, across developmental, evolutionary, and cognitive biology. I then focus on dissolving the binary distinction between agents and the thought patterns within their cognitive system, focusing on the patterns themselves as having an important degree of not only ontological realism [29] but also agency [30]. In this sense, the focus on the dynamics between memories *as agents* and cognitive systems is consistent with several other concepts: radical embodied cognitive science [31,32], the importance of action perception during evolution [33], the idea of picturing engrams as dynamic processes, not as fixed traces with clear and unambiguous representations [34,35], and, broadly, the biosemiotics of meaning [16,36,37,38,39,40,41,42] among and within agents.

Using the lens of memory, and specifically the transfer and remapping of memories across space, time, and Selves in very diverse biological examples, I develop two key ideas. First, that evolution makes problem-solving agents, not solutions, and that its commitment to the reality of mutation and uncontrollable environments—to the active reinterpretation of information on the fly—is a ratchet that gave rise to intelligence and cognitive Selfhood^5^. Second, that the Self is a dynamical construct, not in the sense of a misleading illusion to be eradicated, but in the sense in which all models are compact perspectives created and continuously maintained^6^ by agents under energy and time constraints. Viewing the Self as an intelligent data pattern, which facilitates its own transformation [53,54], is a helpful construct because it captures a core truth: while the details of minds and bodies change, thought forms (salient cores abstracted from experiences) *remain and survive* because they move across architectures that are able to interpret them in new ways for sense-making as a gestalt [55,56,57], rather than committing to their details. I trace back the origin of this property to the ubiquity of polycomputing^7^, and propose the hypothesis that these dynamics enabled the evolution of minimal agency in the thought forms themselves: in other words, giving up the binary dichotomy between computational machinery and passive data, and exploring the idea that certain kinds of information structures *actively facilitate* their transformation and remapping in ever-changing cognitively excitable media. As William James proposed, “thoughts are thinkers”^8^.

## 2. Background: The Shifting Sands of Selves and Memories

*“The material present in the form of memory traces being subjected from time to time to a rearrangement in accordance with fresh circumstances—to a re-transcription.”*

Sigmund Freud writing to Wilhelm Fliess on 2 November 1896

 

Confabulation is a kind of cognitive plasticity that emphasizes the present, the future, and the gestalt over the literal past—it occurs when a mind actively modifies and fits its beliefs to a current context, altering and reinterpreting memory data as needed to preserve various psychological elements in the story that it tells to others and to itself. For example, a patient with an electrode in their brain who involuntarily laughs when the clinician unexpectedly triggers the electrode will often explain this odd behavior by saying “oh I was thinking of a funny joke”; they do not say “Weird, I was thinking of serious things and my mouth started laughing” (because this undermines their self-model as an autonomous agent) [59,60,61]. The same thing happens with split-brain patients, whose left hand moves in ways unexpected by the verbal mind—the language center does its best to explain what has happened by making up stories^9^. This is true not only with respect to high-level thoughts, but even for very basic aspects of our perception: the reason we do not see the big gap in our retinas occupied by the optic nerve is that the brain fills it in—a controlled hallucination or “inpainting” of content that we could not see, based on the surrounding information^10^. This process not only edits the current data, but also those of the past and future. If two light bulbs, yellow and blue, are lit in rapid succession, subjects not only see the light moving but actually report it going through an intermediate green color and position—not only does our brain invent a new color and light we did not see, but the experience is felt out of sequence, perceived as if the inserted information was back-dated (because it cannot know to make the middle “dot” of green until it has actually seen the blue one), editing the memory stream to insert the information in an Orwellian scheme to present a coherent story [64,65]. There are also many examples of anticipation and prediction modifying the interpretation of incoming stimuli [66,67,68] and long-term memories [69]. More generally, Hoffman’s work on the interface theory of perception illustrates how little emphasis evolution places on veridical perception [70,71,72].

**Figure 1:**
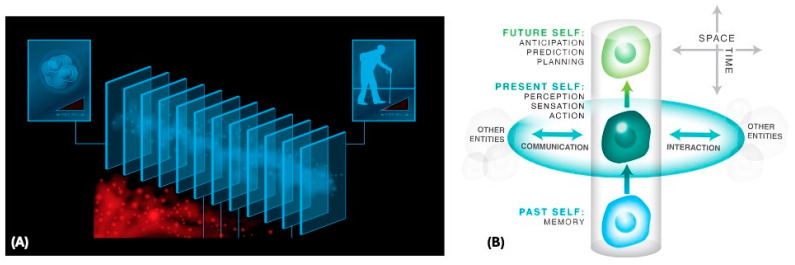
The temporal slices of a continuous being over time (**A**) support memories as messages that pass between the temporal slices (Selflets) of that being, analogously to the messages that pass laterally between different beings at a given time (conventional communication) (**B**). Images used with permission from Jeremy Guay of Peregrine Creative. Note that it is not claimed here that this is *the* correct way to think about Selves—this schematization focuses on an external (3rd-person) perspective that helps in understanding certain invariants in how biology uses information. This view omits the complementary perspective of the experiential Self (1st-person experience of the persistent flow itself).

These dynamic features of our mind^11^ soften our connection to the ground truth of what actually happened in our history [73]. The fact that we cannot really trust our memories of the past—in a much deeper way than just an inability to remember some details—points to the disorienting fact that we do not perceive the past as it was but rather modify and rebuild our mental model dynamically [65,74]. We have no direct access to our past—at each present moment, to recollect the past we have to *reconstruct* it dynamically from the engrams [75] left in our brain (and body) by the activity of a past Self. One can think about our apparent continuous stream of cognition as a series of frames or Selflets (like slices in the bread-loaf model of Special Relativity), each one being probably a few hundred milliseconds thick (Figure 1).

From this perspective, memories are messages between agents separated across time—each engram is a stigmergic [76,77] note left in our body by a past version of us^12^. A view of temporal (vertical) memories as communication between our Selflets invites us to think of memory as parallel to the horizontal communication we do with *others’* Selflets^13^. And like all messages, they need to be interpreted^14^; indeed, von Foerster [82] emphasizes the symmetry between foresight, hindsight, and insight: the key is not the passage of time but rather the need to make sense of the future and the past equally. Memories are also an answer to “what it’s like to be past-me”, in the sense of Nagel’s “what it’s like to be a bat” [83]—we cannot actually *be* our past Self, but we can try to reconstruct it based on the evidence provided by reinterpreting the clues left by them, just as we do for the heterophenomenology of other minds [84]. The task of finding optimal meanings for memory traces is an exercise in intelligence and satisfaction of epistemic hunger—squeezing stimuli (whether from the environment or from one’s past Self) for actionable wisdom about what to do next in novel scenarios^15^.

All this is often considered an unavoidable but undesirable bug in cognitive architectures: confabulation in AI, the notorious unreliability of court witness testimonies, and Humean philosophical arguments (e.g., Boltzmann brains, Descartes’s deceptive demon, etc.) seem to undermine the solidity of our personal identity^16^. They are disturbing when people first hear about them^17^. Here, I argue that this is actually a powerful and important feature, arising from our ancient origins at the dawn of life and helping us to understand many phenomena beyond brains and behavior. The pejorative sense that “confabulation” has today should be revised in favor of a recognition of the importance of sense-making and commitment to adaptive function in the future (over fixed meanings of past data). I propose that the necessity for mnemonic improvisation—the active rebuilding of the content of any (proto)cognitive system—was the source of morphogenetic robustness and eventually conventional intelligence. The ability to improvise and make sense of your world in real time and the commitment to change (not just to persistence) over an allegiance to the details of a past history form a fundamental biological^18^ strategy deployed at many scales, with massive impact^19^.

## 3. Remapping Memories: Beyond Storage and Simple Modification

*“No man ever steps in the same river twice. For it’s not the same river and he’s not the same man.”*

Heraclitus

 

Consider metamorphosis. Caterpillars largely disassemble and remodel their brain in becoming butterflies. Their hardware is strongly refactored as the creature shifts from a soft-bodied machine that lives in a 2D world and eats leaves to a hard-bodied one that flies through 3D space and drinks nectar. The controller is replaced, but some memories are known to remain [26,27,88], and this has also been studied in other model systems including vertebrates [88,89,90,91,92,93]. It is tempting to focus on the important question of “where is the memory stored during this process?” (of course modern neuroscience has been studying the dynamic, complex process of memory formation, encoding, and storage as consolidation)—but there is an even deeper issue here, concerning the encoding, decoding, and *interpretation* of the memory engrams.

Specific memories useful to the caterpillar—for instance, what actuators to fire to make use of the fact that leaves are to be found with a specific color cue (e.g., associative memories cued by a bright red disk)—are entirely useless to the butterfly. The butterfly’s body is controlled very differently—soft-bodied robots need a different controller than ones which have hard elements that can be *pushed* during actuation. Its visual system is different, and most of all, it does not eat or care about leaves—it wants nectar. The process of memory and recall not only functionally generalizes (abstracts^20^) the information (not leaves, but “food”), but most importantly, *remaps*^21^ it in a way that the basic relationship learned maintains its salience in a new body and environment. Stated another way, the being that radically changes does not bring with it specific memories into its new, higher-dimensional life—it brings the deep lessons of its experience in a way that it can deploy in its new embodiment.

Metamorphosing insects and planaria that maintain memories and re-imprint them from tail fragments onto a newly regenerating brain, and anterograde amnesia patients^22^ who update external notepads to keep track of their life—all seem like extreme examples or rare outlier cases of weird memory dynamics. But to various degrees, we are all undergoing this process of sense-making through the improvisation and decoding of clues. Ricard Solé calls ant colonies “liquid brains” [98] because their subunits do not keep a stable connectivity pattern (like a solid) but rather shift around during the cognitive life of the colony mind. Ant colonies are liquid in terms of the spatial positions of the ants, but we are all brains that are liquid *in time* because of the shifting relationship between our Self-model and our cognitive content across moments. The mental content of each Selflet is not welded to that of the previous one in a linear, fixed pattern; it slides around, influenced^23^ by the content of past slices but not over-committing or taking them too seriously^24^ as they reinterpret them to suit new circumstances.

Even mammalian brains change over time. The hormonally driven brain maturation of puberty may not be as drastic a change as that which happens to a caterpillar^25^, but it is significant enough to strongly re-model our preferences and priorities. On a small scale, our brain and body are constantly turning over in terms of molecules and cellular components (including dynamic synapses), and yet centenarians have functional memories from their childhood. Critically, normal recall in the brain^26^ is not a pure “read” operation—accessing a memory changes it [105], which is a fact exploited by therapists treating trauma and by everyday tasks involving creativity and problem-solving [106]. This also underscores the continuous, dynamic nature of memory. This issue concerns much more than robustness—it is not about finding a way to lock a memory in place and maintain its details against noise and perturbations^27^, but rather it is about being able to remap, adapt, and improvise to extract the salience of memories into new contexts. Ubiquitous confabulation [62,108,109,110], and not just the stark examples seen in split-brain patients whose language center concocts a story about why their left hand was moving in surprising ways, is the central invariant across numerous phenomena in evolutionary biology, developmental biology, and neurobiology.

The considerable literature on (behavioral) memory transplants across animal bodies reveals that the ability to reinterpret memories functions between Selflets that are not somatically contiguous (not part of the same organism persisting through time). The results observed when pieces of, or extracts of, the brains of trained animals are moved into naïve subjects [111,112,113,114,115,116,117,118,119], and the (as yet uncertain) reported claims of memories transferred via heart/lung transplants [120,121], point to an even deeper kind of remapping capacity. First, the movement of memories via brain extracts [113] does not require careful placement—in David Glanzman’s experiments, for example, they inject the extract into the general vicinity of the relevant Aplysia nervous tissue. It is remarkable that no matter where precisely the molecules go, the new brain will extract their meaning and instruct the behavior of the recipient Aplysia.

Also remarkable^28^ is the observation that one can inject an odorant molecule into a frog egg, and the resulting animal will seek out that odor in its search for food [122]. Consider what this must mean—mechanisms *inside* a single cell have to extract information from this new input, and convert it into the processes needed to modify the genetically encoded hardware of the nervous system to result in a new behavior that is functionally tied to the sensing of that molecule. Here we have the following: the detection of novelty (how does it know to pay attention to this molecule out of millions of other, native, molecules inside the egg?); remapping across scales (from a single cell to the whole organism); and the preservation of salience across novel scenarios (from biochemical signals in cytoplasm to the dynamics of nervous system function in guiding food-seeking behavior). The engrams seem less like encoded memories^29^, and more like a kind of prompt, with a lot of the hard work being carried out through the encoding/decoding process, which adds interpretation, context sensitivity, and generalization (i.e., intelligence) to the process.

## 4. Beyond the Brain: Bowties Everywhere

*“The past is a foreign country; they do things differently there.”*

L.P. Hartley

 

I have argued elsewhere [96,123,124] that there are profound symmetries between the collective intelligence of neurons in navigating 3D spaces and that of non-neural cells navigating anatomical morphospace. Many algorithms, competencies, failure modes, and molecular mechanisms are shared between the self-assembly/repair of the body and the emergence/maintenance of the mind. This symmetry allows us to think about the relevance of the memory-remapping concept beyond the brain and behavior, and of sender–receiver models [37,78,79,80].

**Figure 2:**
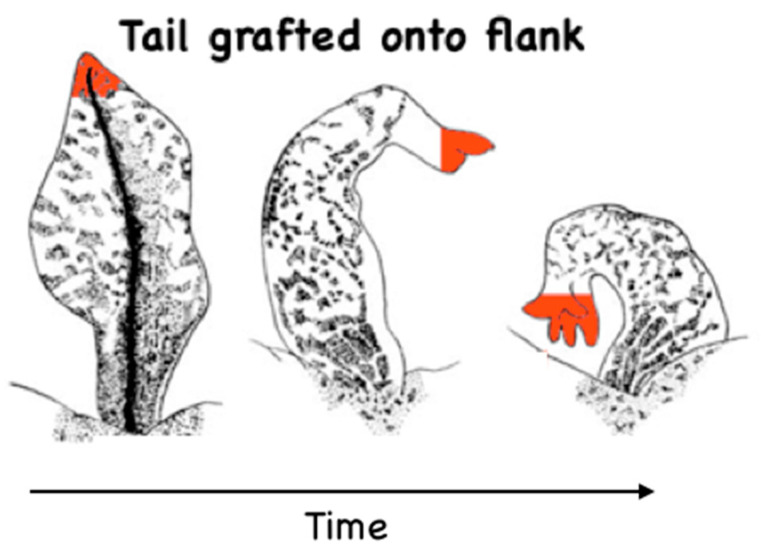
Remodeling of a transplanted tail into a limb-like structure in a salamander. Image from [127].

The first concept that carries over naturally is the notion of triggers—low-information-content stimuli that kick off complex, spatiotemporally appropriate responses because the receiver is, to a degree, intelligent^30^. A simple voltage state imposed on somatic cells can induce them to build a complete vertebrate eye [100]—but of course, that simple biophysical state cannot contain all of the information needed to make an eye^31^. Another voltage-modifying reagent (Monensin) triggers tails to grow in tadpoles, but legs to grow in froglets [125,126], and never vice versa. Here, the same bioelectric stimulus is functionally reinterpreted based on the specific context, preserving salient, high-level information like “build whatever normally goes at this location” (in the case of the appendage regeneration trigger). Remapping is especially salient in the regeneration of organs by bioelectric patterns that are much bigger (e.g., in the case of a planarian fragment that obeys the bioelectric pattern memory of the original body^32^, but only carries a small part of it when it is cut out), or those which are present in tissue that is very different (e.g., a salamander arm that regrows from a body that is missing those structures and has to thus source its information from other parts of the body). The importance of the gestalt and the functional coupling from the large-scale target morphology information through to the molecular events inside cells are also seen in the example of tails transplanted to the flank of a salamander, which gradually remodel into a limb-like structure [127,128,129]. This transformation includes the cells at the tip of the tail, which are correct in their local environment but must change to fit the global pattern (Figure 2).

**Figure 3:**
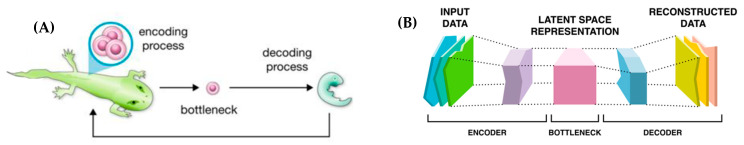
Bowtie architectures feature a low-dimensional compressed medium at the center through which information must come, and active decoding and context-sensitive interpretation on the output. (**A**) Developmental processing of morphogenetic information. (**B**) A typical machine learning architecture. Images used with permission from Jeremy Guay of Peregrine Creative.

Another aspect of this is the compression/expansion cycle of metazoan organisms. Each organism does not make a copy of itself—it undergoes a massive compression, producing an egg, which then has to re-inflate during the process of self-assembly and engage with a possibly different world using possibly different (mutated) parts (a kind of de-generalization, to apply past patterns of wisdom to novel scenarios). Some somatic information does not make it through, but it is increasingly recognized that a lot of generic states (like “stress”) does [130,131,132,133,134,135,136,137]. The importance of a process that squeezes down complex states into a compact, generalized representation (a so-called bowtie architecture of information, as seen in Figure 3) is routinely used in AI (the autoencoder).

Tadpoles produced with no endogenous eyes but bearing an eye placed on their tails can see quite well—they learn effectively in visual assays [138]. These ectopic eyes connect to the spinal cord (not brain), or sometimes the gut. Why does it not require many generations of mutation and selection to enable this new sensorimotor architecture to work effectively? This kind of plasticity is remarkable on the one hand, as we expect the lessons of evolutionary history to be passed down in a deterministic fashion to the next generation. On the other hand, we can consider development as an instance of regenerative repair, with each developmental stage being a birth defect relative to the next stage that must be dynamically repaired as the organism progresses through maturation. Regeneration in adult salamanders, of a limb from the rest of the body, is not nearly as impressive as the trick that *all* metazoans do—regenerating the entire body from one cell (including mammals, normally thought to not be regenerative).

**Figure 4:**
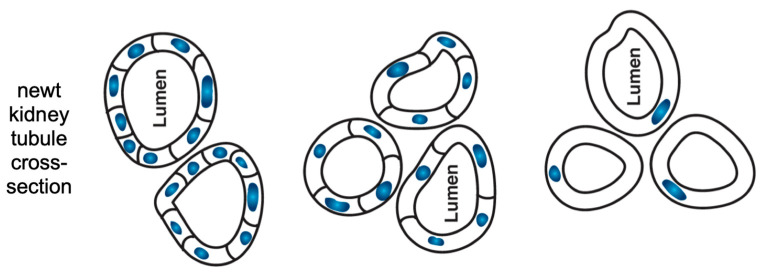
Cross-section of newt kidney tubule at different ploidy levels. Re-drawn by Jeremy Guay of Peregrine Creative from [142].

Regeneration of limbs and eyes is not surprising when one considers that the organism has to be able to construct itself from one cell, or from a mixed-up collection of cells^33^. But it is more than uncertainty of the external environment that limits the relevance of past generations’ specific lessons: it is also uncertainty about one’s own parts. Engineered polyploid newts complete normal development despite their much larger cells and extra copies of chromosomes [142,143]. Their kidney tubule diameters (Figure 4) stay normal, being made of up fewer, but larger, cells. If their cells are made truly enormous, then just one cell will bend around itself to complete the relevant task in anatomical morphospace. This requires using a different molecular mechanism (cytoskeletal bending instead of cell–cell communication of tubulogenesis)—calling up different modular subroutines to accomplish their high-level goal state as needed, while circumstances (indeed, their inner components) change unexpectedly^34^.

**Figure 5:**
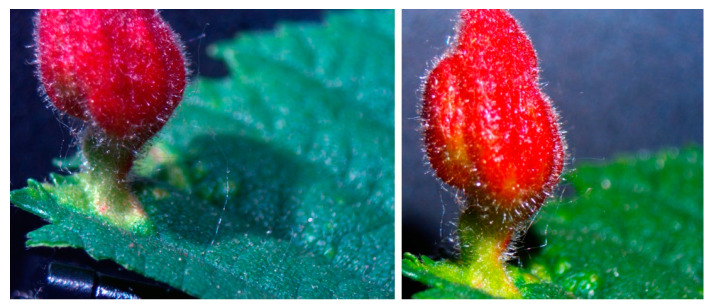
Novel forms made by genetically normal plant leaf cells, when prompted by signals from a wasp embryo.

Think of the task facing such an embryo coming into the world. It cannot take for granted how much genetic material it will have [143], or how many cells [146,147,148], or what size cells [142], it will have (in addition to any external damage it might sustain, or the chemical details of its environment—even the internal parts cannot be assumed to be the same as those with which its past genome was forged). Beyond the incredible robustness of standard form and function, we see adaptation to extreme changes of conditions, with no need for transgenes or genomic editing, in the spontaneous emergence of Xenobots [149,150,151], Anthrobots [152], and plant galls [153] (constructions of leaf cells hacked by signals from a non-human bioengineer to produce a totally new and complex pattern, as shown in Figure 5).

**Figure 6:**
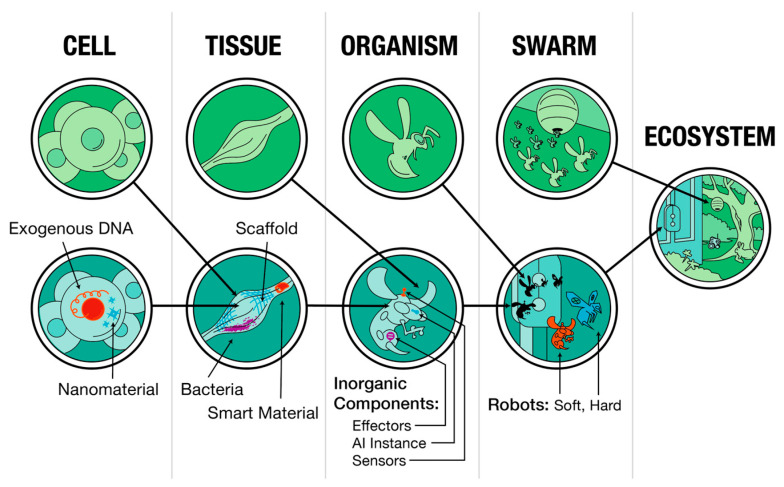
A schematic of how biological systems can adaptively incorporate foreign material (whether evolved or engineered) at every level of organization. Taken from [124]; image by Jeremy Guay of Peregrine Creative.

This is also probably why chimerism and bioengineering, at all scales, work—coherent outcomes often result from combining not only divergent living components from different lineages [154] but also totally unnatural and novel engineered components, from nanomaterials to smart implants [155,156,157,158,159,160] (Figure 6). Biological components are primed to accommodate and exploit whatever materials and processes are in their vicinity (as useful functionality and computations).

We do not know how this remarkable plasticity works yet, but one thing it does, to some extent, is free the downstream (future) agent from the restrictions of the upstream (past) agent. If we consider genomic DNA to be the engram^35^ of the evolutionary-scale individual (the entire lineage as one huge time-extended agent), then its interpretation and usage occurs via remapping and repurposing as needed, just as conventional memories do in smaller-scale individuals. The hourglass architecture is extensively used in technology and can be used to understand modularity and anti-fragility in both designed and evolved systems [162,163,164].

**Figure 7:**
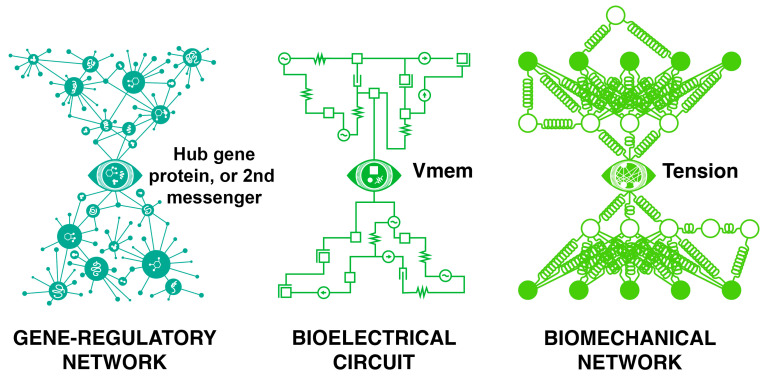
Schematic representations of bowtie architectures in biochemical, bioelectrical, and biomechanical circuits. For example, causal parameters such as tension or resting voltage potential across the membrane (V_mem_), as well as specific genes, can act as information hubs that ensure the compression of signals within biological control networks.

Note that this bowtie architecture—which *forces* a compression^36^ of data to a generative kernel that then has to be re-inflated and elaborated—is also a common feature of biochemical, bioelectrical, and biomechanical pathways (Figure 7), that is, at the subcellular scale; at the super-organism scale, recent data also reveal that a very simple parameter—waves of ATP—can mediate the complex morphogenetic information being shared and processed within *groups* of embryos^37^ defending their anatomies against teratogenic influence [167]. The same architecture can be used to think about learning (when one side of the bowtie is the environment), training (when the environment contains agents with agendas that send messages with the goal of changing the behavior of other agents), and other kinds of communication both laterally and vertically (to one’s future self).

One of the most interesting things about compression, such as that seen in learning and generalization, is that by removing correlations, the resulting engram looks increasingly random^38^. Because there is no “outside-text” or meta-data [168], the interpretation by the right side of the bowtie (or the future Self) must be creative, not only algorithmically deductive, in interpreting it in future contexts (as both the environment and body internals shift). The sense-making process of memory interpretation and the formation of models representing internal states and the external world is creative as much as it is driven by information processing and past data. As John Truby notes, “a great story is organic—not a machine but a living body that develops” [169]. In this sense, the development and regenerative maintenance of a body through morphogenesis, guided by biophysical models [170,171,172,173], is a kind of dynamic elaboration of a story (about anatomical morphospace).

What underlying parameter is represented by the spectrum ranging from the hardwired, mosaic *C. elegans* to the intermediately plastic amphibia and (embryonic) mammals, and to the extreme plasticity of planaria? I propose it is the *willingness to confabulate in anatomical space*—a pattern extraction and completion^39^ architecture that does not take priors too seriously and assumes that it will have to develop models of itself, its problem space, and its internal and external affordances on the fly (a kind of “beginner’s mind”, emphasizing forward-looking creativity over past-constrained structure). This suggests a strategic parameter for morphogenetic/cognitive systems akin to “r vs. K” selection in evolutionary ecology [175]. In silico simulation results [176] show how this kind of process can give rise to remarkable lineages, such as planarian flatworms, which are extremely resistant to transgenesis, aging, cancer, and injury despite^40^ their incredibly noisy genome because they have fully committed to a strategy that overrides genetic details with large-scale pattern completion [176].

## 5. Beyond Biology

This idea of complex data acquiring robustness by being squeezed into a bowtie bottleneck can be found outside the examples of development, evolution, and behavior (see examples in Table 1). For example, communication between two humans can be seen as the bowtie scheme, with language as the hub [178]. We cannot simply exchange tables of neuronal state data with other humans, animals, or AIs—those details cannot be remapped from brain to brain directly. But, squeezed down into the low-bandwidth channel of language, the salient aspects of the message can be remapped by the listener’s brain into whatever biophysical changes are needed to implement the interpretation that makes the most sense to them^41^ [179,180].

Similarly, the scientific research process can be seen this way. Scientists accumulate a coherent world model after years of experimental observations and analysis, and present it as a “published manuscript”—a low-bandwidth, impoverished stimulus from which other scientists will extract information that may change their own world view and mental content (and maybe in ways other than what the author intended). Poetry and art achieve much more drastic encoding/decoding dynamics when they pass from artist to viewer^42^, requiring massive amounts of personal interpretation and modification on the decoding end of the recipient, while baking recipes require less.

**Table 1:** Diverse examples of information compression and reinterpretation.

Scenario/Scale	Bowtie Hub Node	Remapping Process
Developmental lineage	Egg	Morphogenetic problem-solving competencies
Stress	Integrated stress response	Multiple physiological systems performing credit assignment to adaptively adjust to general stress indicators
Hyper-embryo groups [167]	Calcium/ATP signal through the medium between embryos	Increased morphogenetic problem-solving competencies
Hologram^43^	Holographic film, storing a compressed complex 3D pattern in a 2D substrate	Laser interrogation
Regeneration	Bioelectric pattern	Planaria remapping V_mem_ map from whole to fragment
Single cognitive Self across time	Memory media (engrams)	Neural interpretation of engrams
Transplants between cognitive Selves	Extracts (RNA) or tissue implants	Neural interpretation of engrams
Communication	Language [185]	Neural interpretation of spoken/written messages
Psychoanalysis	Dreams, speech acts	Creative, intuitive, skillful interpretation for a therapeutic goal
Song	Written musical scores	Replaying the same song on a totally different instrument
Science, in the short term	Talks/manuscripts	Interpretation of data by scientists in the same/other fields
Science, in the long term	Ideas and paradigms, explanations	Interpretation and use by the scientific community: some ideas become immortalized as engineering tech; others are revised, or forgotten.
Art	Poetry, paintings, etc.	Personal interpretation and finding personal meaning
Society	Religious frameworks	Adapting (or not) as technology and science advance

There are possibly implications here beyond conventional third-person science. First, a commitment to meaning and interpretation could potentially be useful in a psychological setting, whether personal or therapeutic, in the sense that techniques could be developed to help reinterpret somatic and cognitive memories of past trauma^44^. In the short term, two individuals may see the same event (e.g., chopping down a tree) very similarly and agree on the micro details of what they saw. But on a longer timescale of interpretation, each Self views this differently (perhaps, as a value-creating progress vs. the destruction of an ecosystem)—the memory is embedded in a different mental structure in which the meaning of the event (and thus its contributions to future thought processes) is private and unique, while the details were the same. The answer to “what did you experience?” is critically tied to the timescale on which the answer is based.

## 6. What It Means, and What Next: A Research Program for Further Development

*“A story has no beginning or end: arbitrarily one chooses that moment of experience from which to look back or from which to look ahead.”*

Graham Greene

 

The basic fact that the species with the noisiest genome is also the species with the best regeneration, immortality, and cancer resistance, which has been a puzzle [177] for a century, is rarely talked about within a paradigm in which genes should encode, and determine, phenotypes. It seems that the underlying noise and unreliability of biological matter is not a bug but a feature: we obtained morphological (and behavioral) intelligence because of, not in spite of, the vulnerability of the substrate^45^. The competency of our material means that selection cannot see the quality of genomes clearly^46^. More evolutionary work thus has to be conducted on the competency mechanisms, not the structural components, leading to a positive feedback loop—an intelligence ratchet based on the remapping of information to new contexts^47^. It is an important direction for future research to understand what factors determine where on the plasticity and intelligence spectrum any given species comes to reside (with respect to morphological, behavioral, biochemical, and other problem spaces).

While our engineering efforts build computational algorithms on top of extremely reliable hardware, life instead doubled down on the “play the hand you’re dealt” strategy, not over-training on evolutionary priors and committing very early to sense-making at all scales. And this not only concerns the robustness of the hardware; it is about the semantics of the software and its data. Current computing architectures view data as a passive object to be interpreted in one way, from the perspective of the user—it is the job of a conventional computer to facilitate that one interpretation and faithfully *store* and transmit the data^19^. Biology uses a polycomputing architecture [58,209], committed to on-the-fly confabulation, in which a heterarchical soup of competing, cooperating, multi-scale agents vie to develop viewpoints from which the molecular and biophysical states are interpreted (and hardware affordances are hacked and reused [31,210,211]) in whichever way they best can be at the time^48^.

One major area for future work is the nature of the memory medium and how it facilitates reinterpretation and invariance of saliency: if engrams are not in the synaptic structures [217], where are they? My current hypothesis, driven by the above multi-scale perspective [218], is that there is *no* single substrate for memory^49^. Every component of the system, including but not limited to those that bubble up to conscious recollections, could be using everything in its environment as an interpretable scratchpad^50^. The deep levels of biological structure and dynamics offer an incredibly high-dimensional reservoir [223,224,225,226] (referred to as the senome in [19,227]) that can be exploited for memory remapping^51^. In this view, neuronal networks are not so much used for holding memory as they are for learning to interpret the engrams embodied by subcellular components [34,35].

An immediate opportunity for future development concerns the mechanisms and algorithms of informational remapping. By modeling networks of self-organizing observers (and tracking the emergence of autopoietic *perspectives* [230] and polycomputing competencies), we may arrive at testable hypotheses about how memories can be remapped to new contexts. Reservoir computing [223,224,225,231] may also offer interesting tools for understanding how rich biological structures can be interpreted on the fly. Model systems for research on moving memories range from in silico systems (the transfer of learned memories across gene regulatory network models with different geometries) to in vitro/in vivo ones, such as the movement of pattern memories from two-headed planaria to wild-type hosts via tissue implants (unpublished preliminary data on this already exist), and the movement of behavioral memories (such as nicotine addiction) from human donors through Anthrobots to rat hosts in which we can implant them (with the question being, will they self-medicate?). The lessons we learn will likely be actionable for making new kinds of truly bio-inspired AI architectures [232], and will also help design strategies for regenerative biomedicine that target how cells and tissues interpret the interventions we provide in the form of biochemical and bioelectrical stimuli [123,233,234].

Computational models of this process can investigate whether, for example, spatial information can be remapped into temporal information. Some great work is being conducted in this area in the field of Artificial Life, starting with the original Bongard experiments of a robot that did not know its shape and had to discover it, and then re-use that information when its body shape was abruptly changed due to damage [235], and other work on the transfer of skills in dynamical recurrent neural networks [236] or deep learning in embodied robotics [237]. Another possible model system is that of sleep, and the difficulties of the waking self to make sense of the memory traces left by the sleeping self. Perhaps some of the tools of dream analysis could shed light on the more generic memory reinterpretation process^52^.

## 7. A Continuum: From Thoughts to Thinkers

*“We are pleased to have helped you. Goodbye.”*

a hallucinatory voice which correctly diagnosed a patient’s unrecognized brain tumor [242]

 

A specific hypothesis that can be investigated is the extent to which memories, especially non-local ones, actually reify the agent to whom they belong, in a sort of feedback loop in which the Self elaborates and maintains memories, which in turn reinforce its existence beyond that of its parts. Can they serve as one of the cognitive glue mechanisms that makes the whole more than the sum of its parts? For example, consider a rat trained to associate pressing a lever with receiving a reward. No individual cell has both experiences—the skin cells of the paws experience the lever, the cells in the gut receive the metabolic reward. This associative memory belongs to the “rat”—the collective—and to none of the cells individually. Could having such memories actually help create the organism in a real sense? The body houses and maintains them, but perhaps they in turn make the virtual governor [243,244] that we call the cognitive Self *more real*. This is currently being investigated in the context of our training of associative memories into gene-regulatory networks and other substrates, with measures of integrated information to determine if the forming of memories increases the self-coherence of composite systems. Many such experiments suggest themselves.

Furthermore, more broadly, could we blur the boundary between passive data and the active cognitive architectures that hold them—between thoughts and thinkers? For example, it has been argued that what persists are *algorithms* [245], which is a powerful way to think about active information. However, what if we go further on the continuum, beyond passive and even active data, to basal agency? Perhaps there is no principled, sharp distinction between data and algorithms, between memories and minds—but rather just a continuum of different degrees of agency between the understander and the understandant [246]. This would also require a continuum between skills (“knowing how”) and propositional knowledge (“knowing that”). What if, in James’s words, “thoughts are thinkers” in the sense that they actively help (perhaps by cooperating and competing for opportunities^53^ or using each other as affordances in a heterarchy) cognitive systems to remap and utilize them^54^? What if memories, which are not static details but active deep patterns, can resonate^55^ with a cognizer or even a group of cognizers (in the case of federated inference and belief-sharing [251]) in a kind of circular causality [252,253,254], in which they exert some minimal agency as they shape the mind of the thinker and thus help construct the niche^56^ within which they will be utilized in subsequent time steps^57^?

From this perspective, the continuum can range from fleeting thoughts, to persistent/intrusive thoughts, to the kinds of metastable entities experienced in tulpamancy [256,257], to dissociative [32,258] and other kinds [242] of alters, and finally to conventional full personalities (minds) that can generate all of the prior members of the hierarchy. It seems crazy to think that an agent, even a minimal kind, can be just a metastable pattern in an excitable medium—a temporarily persistent pattern^58^. But that is what we are too—temporarily persistent, autocatalytic, dissipative patterns that self-reify our boundaries from the outside world via active inference and interpreting our environment to tell coherent stories (models) that hold us together and make us more than the sum of our parts [260,261,262,263,264,265,266,267,268,269,270,271]. And on an evolutionary scale, the thoughts of a lineage mind, of which each individual creature is a hypothesis about the outside world, are definitely active agents (they are the conventional, medium-scale agents we recognize every day as behaving life forms).

Thus, there is the possibility to develop a rigorous model for how thoughts can scale up to become thinkers of their own^59^, spawning off new thoughts as a virtual stack of resonances (perhaps by closing some “strange loop” [54] of self-reference and self-reification). But, on a more practical level, these ideas could be tractable now in a biomedical context, to develop tools to model, recognize, and control persistent physiological patterns that represent stresses, priors, setpoints, etc., as basal thought forms in the mind of the collective intelligence of the body operating in physiological, metabolic, and transcriptional states. It is not hard to imagine how managing such self-perpetuating states could be useful for biomedicine [104]. Emerging tools of 4D physiology, in vivo imaging, and optogenetics are currently being used to test these hypotheses.

Although a full account will be given elsewhere, it is tempting to mention the possible links to consciousness. Could consciousness simply be what it feels like to be in charge of constant self-construction, driven to reinterpret all available data in the service of choosing what to do next? In this sense, cognition is essentially freedom from the past; cognitive Selves could be systems that are not committed to their own past and their own memories. Paradoxically, biological Selves do not take themselves too seriously in the sense that they are not committed to a fixed set of meanings established by their prior Selflets—their freedom consists not only in actions, but in forward-looking sense-making of their own mental content. Letting go of the past Self and living life forward is a commitment to making the best of internal, not only external, information. This is in broad agreement with Solms’s idea that consciousness is palpated uncertainty about the outside world [67,272]; I propose to expand this idea, with the hypothesis that consciousness is palpated uncertainty about your own memories and internal states.

## 8. Conclusions

*“Now I do not know whether I was then a man dreaming I was a butterfly, or whether I am now a butterfly dreaming I am a man. Between a man and a butterfly there is necessarily a barrier.”*

Chuang Tzu

 

This work is a continued exploration of classical ideas in which the construction of the mind and that of the body are tightly linked [56,57,273,274,275,276,277]. Future work will explore further the implications of this perspective for the ontology of patterns as agents [29,30], for efforts to unify semantic information and agency in a physics framework [246,278,279,280,281,282], and for panpsychist views of the mind–matter relationship [283,284]. Here, I have tried to provide the strongest versions of claims along these lines, to help crystallize how far these ideas could possibly go and what they may imply. It is entirely possible that the most useful lens on the topics discussed above will turn out to be a more moderate version—an intermediate hybrid between the conventional picture and the full-bore picture proposed here.

Organisms that cannot handle novelty—of their own parts and memories, not just of their environment—will not be evolvable and are likely not to persist. The symmetry of precariousness and uncertainty in the environment and in the internal milieu underscores the tenuous line between organisms and the outside world—both are mysteries that must continuously be grappled with, by life’s essential adaptive sense-making competencies. I hypothesize that remapping memories onto a new body (and, indeed, maintaining memories across a lifetime) works because development is also regulative and able to remap genetic information into new scenarios, as in the salamander kidney tubule and similar examples. Morphogenetic and cognitive intelligence (problem-solving, adaptive competencies, and plasticity) arise because of the constant need to battle dispersive, degrading tendencies of the material. This cannot be performed in an invulnerable, static substrate but rather requires active reinterpretation in real time [73]. Regeneration, regulative development, hackability, and the interoperability of life in weird new configurations are all consequences of repair and remapping processes. Thus, life, at all scales, from the microscopic to the trans-personal, is all about repairing and defending the Self, because the Self is a construction^60^, in the most powerful and useful sense—an adaptive, actionable, embodied story that holds components together and enables intelligent navigation of a problem space.

Selves are simultaneously a construct in the mind of an observer(/observers), including itself, and real, causally important agents that live, suffer, die, strive, and matter. I think this also helps us in understanding what an observer is [285]. An observer is real and significant to the extent that the content of what they observe makes a difference to them and their future behavior—they will act differently^61^ based on their interpretation of the signals they receive (unlike, for example, a telescope, or even photographic film, which forms a memory record but does not analyze in a way that links up to any cybernetic perception–action cycles). Observers interpret what they sense from their own perspective; their allegiance is to extracting meaning, not preserving accurate details. It is the mark of significant observers that they exert their agency not in seeing an event or a set of states as they are, but rather in weaving a coarse-grained compression that adaptively captures what is sensed in a process of autocrine storytelling that will be easy to exploit in future behaviors. The criterion for being an observer is that an observer is fundamentally committed to reinterpretation and meaning, not micro-scale realism [70,71,72,286]. They bring their own history, perspective, biases, hedonic valence, and predictive coding strategies about what is important in sensory and interoceptive experiences [47,65,67,73,272,287,288]. Currently, only biological beings are clearly recognized to be able to do this significantly, but these capacities do not require protoplasmic substrates or an origin through random mutations, and likely can be engineered in an endless range of novel forms of an embodied mind [98,289,290,291].

Tracking back the causality in this space of ideas suggests the following. Behavioral intelligence in 3D space evolved from morphogenetic problem-solving competencies in anatomical space [97,124]. Those competencies in turn were required by the inevitability of evolutionary and physiological damage (mutations and injury), and the ratchet mechanisms of constructive neutral evolution and the paradox of robustness [203,204,205,206,207,208]. The capacity for creative robustness is implemented by the polycomputing property—the ability to see the same physical process as computing and providing different functions depending on perspective^62^. And that, in turn, comes from our multi-scale architecture: at every level, we are a collection of minimal agents that are all making sense of and hacking everything around them (their parts, their neighbors, etc.) as best as they can to fulfill their minimal but vital agendas [279].

Much of biology and cognitive science can thus be seen from the perspective of this fundamental paradox: “do I still exist if I change”? Creatures, whether biological or technological (or both), that resolve this Zen-like riddle do not just persist—they thrive. The inorganic world, and much of today’s engineering, is stuck in an object-centered, matter-first view [292]. Biology embraces, and has from the start, a process ontology in which perspectives and agency are primary; thus, change is the driver of intelligence, and perspectival storytelling is a primary mechanism through which diverse minds transform and grow. I think the lesson to take from this is to embrace the dizzying freedom of breaking away from the goals and structures handed down to us from our evolutionary and personal past, and take on the responsibility of writing our own, improved somatic and mental patterns and values for the future. What engrams do you want to leave to your own future Self, and to humanity’s collective future? Despite knowing that they will not interpret them in the way you may envision now, it is still wonderous to imagine every act as a benevolent communication event to a future being.

## Notes

1 The below analysis of this problem is performed in the context of the emerging field of Diverse Intelligence, which seeks symmetries among agents regardless of their composition, spatiotemporal scale, or origin (evolved vs. engineered). This field also seeks to understand the relationship between life and cognition; to understand the deep nature of embodied minds beyond contingent facts of evolutionary lineage on Earth [1,2,3,4,5,6,7,8,9].

2 Definitions, for the purpose of this paper: Self = a process by which an emergent perspective on the world persists and embodies, but feels (from the inside, from a first-person perspective) as a persistent structure. Intelligence = a publicly observable (third-person perspective) degree of competency at reaching the same goal by different means as necessary [14], with respect to a problem space and goals identified by some observer (which can also be the system itself).

3 Iain McGilchrist points out that snapshot selves are particularly a “left hemisphere” invention—a framing used because it does not understand flow.

4 This is related to the concept of “precariousness” in the enactive cognition field [18] and to ideas on the relationship between the different kinds of disorder and death in living systems and the need for freedom from fixed interpretations of the past [19]. Note that this is a matter of timescales, with respect to how quickly various components degrade relative to the role they perform within the agent. See [20,21,22] for more on the relationship between vulnerability, agency, and plasticity.

5 This idea is also discussed with respect to intelligence and uncertainty in [43], with specific ideas around phase codes and tensors as the mechanisms. There are also other candidates for such over-arching concepts, including the resolution of ambiguity [44] and free-energy minimization [45,46,47].

6 This maintenance requires constant adjustment of the estimated boundary between the Self and the world, and of the sensors and effectors an agent thinks it has. The plasticity of this process, and its lack of allegiance to evolutionary priors, is revealed for example when humans, having spent millions of years of their history as a tetrapod, adopt a new limb as their own within minutes of experience in the “rubber hand illusion” experiments and other cases of sensorimotor augmentation [48,49,50,51], or in syndromes where patients deny that their conventional bodyparts belong to them [52]. The ongoing maintenance of the cognitive Self is highly analogous to the ongoing maintenance required of the somatic self, as the Ship of Theseus of the body resists aging and degenerative processes while remodeling as needed to fit environmental demands.

7 Polycomputing [58] is a kind of observer-first way to see living systems, in which each subsystem can and must interpret the actions and outputs of their neighbors and their own parts. In this framework, computations do not have a single, objective, correct meaning: the same set of physical events can be seen as performing different computations by different observers. By focusing on different ways to interpret the same processes, biological subsystems can adaptively make use of each other; within a body, this allows evolution great freedom to innovate—adding new functionality by adding new interpretations of mechanisms, rather than having to change those mechanisms and risk ruining all of the other systems that depend on them (thus avoiding catastrophic forgetting—a problem that plagues machine learning—and preserving hard-won gains). This freedom from how others in the past intended something to be used or understood is an essential component of the notion of *hacking*, which is ubiquitous in biology.

8 This can be further unpacked into a multi-scale vision of endless stacks of virtualization, where we—as patterns—have thoughts, and thus thoughts can have thoughts of their own.

9 This is also true for intact individuals, who often attribute fabricated reasons for decision-making [62,63].

10 This applies both laterally, and across time (because of saccades).

11 These begin at the very front of the perception cycle, during active processing by the retina.

12 The corresponding memory medium for ant colonies—another collective intelligence—is the ground, on which they leave chemical messages that mediate the colony-level decision-making.

13 Memory is communication, and the mechanisms used to assemble those communications into an emergent Self-model that seems to persist over time is analogous to the mechanisms that bind individual agents (like cells) into an emergent Body-model, because in both cases a continuous stream of signals (passing messages) underlies a temporally and spatially extended whole (as perceived by itself and by external observers). An interesting direction for future research then is to ask what role, within a persistent cognitive Self, is played by the competitive and cooperative dynamics studied in evolutionary game theory within communication between agents [37,78,79,80].

14 “The Mind is Flat” [81] is an excellent discussion of how this happens in real time.

15 The model of intelligence implemented as the analogy-making Copycat system [85] is a great example of this: learning that “abc” maps to “123” will later have to be used to guess what “def” (or even “deg” or “456”) maps to, and it is not the original stimulus but rather the extracted mapping that has to be stored and applied as best as one can to a future situation.

16 This is because our memories are the reference point for everything else we think we know about ourselves—if our memories were changed, in a consistent way, we would never know.

17 They can even lead to existential crises—“a thought that breaks the thinker”—for some.

18 There may be a useful definition of “life” that could be developed with respect to this. We certainly do not call unchanging informational media (e.g., a magnetic disk) “living”, and change is not enough to signal the presence of life, but change in a way that preserves saliency for some observer across transformative steps may be a signature of life in unfamiliar embodiments.

19 This may be related to the limits of formal systems with respect to polysemy, and raises the question of how to go beyond these limits [86] in creating Artificial Intelligence systems that are both reliable (consistent) and creative (inconsistent) [87]. For example, GPUs allow for high performance at the cost of nondeterministic results.

20 Von Foerster [17,94,95] talks about the role of inductive inference, and suggests that living organisms’ and human organizations’ efforts to use past experiences to predict the future, and memory, are fundamentally a form of generalization and not inherently temporal. This emphasis on sense-making over time, as a uniquely privileged aspect, is consistent with previous suggestions that evolution readily pivoted the competencies of bioelectric networks’ control of spatial pattern during morphogenesis into nervous systems’ control of temporal patterns during behavior in a 3D space [96,97].

21 In other words, unlike error correction (de-noising), which aims to reconstruct the low-level details of the message, the information is instead reinterpreted as needed to extract useful meaning, with allegiance not to the original sender and their intent, but to the *current* recipient and their perspective. This is related to the notion of universal hacking of biological parts exploiting neighbors and subcomponents [58], in the sense of a lack of commitment to others’ interpretations, and even your own past interpretation (an essential ingredient of a growth in wisdom).

22 Such patients cannot remember a doctor they just met but will refuse to shake hands with him if he poked them with a concealed pin the first time they shook hands, rapidly concocting stories like “I don’t like to shake hands” [63]—an alternate form of memory that also requires confabulation to fit it into their sense of Self.

23 From this perspective, memories can be seen as prompts—just extremely rich versions of trigger stimuli—that push the cognitive system into specific movements in linguistic or behavioral spaces. Similar phenomena have been studied in anatomical morphospace, where simple bioelectric states likewise trigger memories of organogenesis [99,100].

24 There may be parallels here to “over-training”, as seen in ANNs, as well as changes to memory in trauma and PTSD [101,102,103,104].

25 However, the same issue comes up for human practices, leading to self-transcendence.

26 Perceiving pattern memories in morphogenesis (the anatomical collective intelligence) is also not a pure “read” operation, as many biochemical signals are modified/destroyed by receptors that bind to them during cell–cell instructions in the navigation of anatomical space [96].

27 One thing that this model does not yet deal with, however, is exceptional cases of eidetic memory, as well as failures of generalization [107].

28 The injection of odorant molecules into an egg, which changes the behavior of an adult animal [122], is an example of a small information structure taking over the functionality of a much larger whole, with a past history and its own information structures in place. Another example is the induction of ectopic organs by a small region of bioelectrically-modified cells which disagree with the established fate of the much larger tissue environment and induces neighboring cells to change course and participate in the ectopic organ formation [100]. Why does this work? One possibility is that it triggers a built-in drive meant to overcome the dark-room problem of active inference: if nothing new happens in a long time, it is possible that the lack of surprise is due to a maladaptive failure of infotaxis; thus, there may be selection for a module that counteracts boredom with excessive attention paid to any new information that disagrees with the too-successful world model. The ability of tissues to change on a dime with small trigger inputs makes systems hackable (and susceptible to exploitation) but also enables cognition and evolution (mutations, like ideas, can make huge differences). One formalism that might be useful is that of tiling the plane (because of the relevance of *alignment* as cognitive glue), where one abnormal shape nucleates changes and geometric frustration that can spread through the whole system.

29 Von Foerster emphasizes the difference between recognition/recall (a fundamentally computational and dynamic inference process) and mere storage/retrieval [82,95].

30 In this scheme, we can think of the left and right sides of the bowtie architecture (Figure 3) as two agents, having to communicate via a narrow in-between interface, like language that helps complex beings cooperate through a low-bandwidth interface. Being agential/intelligent means that you do not track microstates—the communication channel does not preserve the lowest-level bits, but rather it preserves meaning or saliency. In biology, this is essential because the parts are never guaranteed to preserve low-level details: engineers make cables where the slightest change of pinout can destroy the functionality; biology assumes there will be “pins” missing, reversed, hacked, etc., and that it will have to spend effort to decode and extract meaning not dependent on the microstates of the message. This is why one can inject brain homogenates blindly into a recipient in a memory transfer experiment, and perhaps why you can randomize the pixel elements of a photo of a dog and an ANN can still recognize the dog-ness of it.

31 As Douglas Brash points out (personal communication), re-writable memory could be viewed as the art of building a new triggerable module.

32 The process of planarian regeneration, in which tiny pieces are able to restore an entire animal, is especially reminiscent of holographic properties [43], but all reproduction can be seen this way—restoring the entire body from one egg cell.

33 For instance, the reconstitution of dissociated Hydra [139], the planarian chaotic mode of development [140], Xenobots assembling from dissociated frog embryo cells [141], etc.

34 In effect, this is a kind of top-down causation or pattern completion (low-level mechanisms affected by the higher-level gestalt) akin to predictive processing in cognitive systems [47,144,145].

35 Indeed, one recent paper shows DNA damage and repair in the context of memory formation [161].

36 This enormous compression is achieved because of the intelligence of both sides of the bowtie, and their incentive for mutual understanding. In general, there is a fascinating dynamic here between forces that incentivize clear communication (encodings that are understandable to future instances of oneself) and cryptographic resistance to hacking from future instances of others [165,166].

37 It is possible that large groups work better than small ones for the Cross-Embryo Morphogenetic Assistance (CEMA, [167]) effect because bigger groups provide a more compelling, more fault-tolerant context for interpretation of the simple signals. With increased distance between the individuals, the latency makes it hard for the collective to maintain the bowtie architecture.

38 This is well recognized, for example, by SETI (Search for Extraterrestrial Intelligence) workers, who must grapple with the fact that messages from advanced beings will very likely look very random to us.

39 Pattern completion—filling in gaps (physical and informational) and out-painting—is a key aspect of machine learning and evolution of cognition [174].

40 It is actually not despite them, but *because* of them. Asexual planaria accumulate mutations due to their somatic inheritance reproductive mode, and largely ignore them—evolution put all of its effort into an algorithm that can reliably make and remake a planarian body despite the extremely variable (mixoploid!) and unpredictable hardware. I have hypothesized that this is due to a competency ratchet in which the remapping process hides information from selection, putting all of the pressure onto the competency mechanisms, not the structural genome [176,177]. Because of their chaotic hardware, planaria commit to a large-scale body plan, not the details of the structural genome—an extreme type of morphogenetic intelligence in the sense of “same goal by different means”. They epitomize top-down control and sense-making in the form of extracting high-level wisdom from the low-level details provided by their genomic data.

41 As Chris Fields points out, “this kind of boundary/Markov blanket thinking says that a shared semantics can never be inferred from observations. It turns Wittgenstein’s private language argument on its head—languages are useless if they are only private, but mechanistically they are private. “Shared semantics” is a constantly evolving negotiation between language users” (personal communication).

42 This connects to ongoing debates about the status of an author’s intended meaning vs. that assigned by consumers of their work. We can visualize how semantic autonomy and deconstructive interpretation extends beyond written works to their antecedents—the dynamic patterns inside minds which progressively broaden out and change the more they interact with other agents, and even their author’s future Self, in a kind of (non-quantum, but perhaps related) decoherence.

43 Note the model of the holographic mind [43,181,182,183,184].

44 Perhaps research on psychedelics/plastogens, trauma, and memory are relevant [186,187,188,189].

45 In the same way, reservoir computing needs materials with high degrees of freedom.

46 Much like the eggshell and the egg’s maternal resources protect an embryo from the environment and harsh selection until its maturity, competency protects a fragile genome from selection too (and assimilation may “mature” it).

47 Some of this dynamic is captured by the emphasis of the extended evolutionary synthesis approach [190,191,192,193,194]. This is especially consistent with the concepts of constructive neutral evolution and the paradox of robustness developed by Susan Lindquist and Steve Frank [195,196,197,198,199,200,201,202,203,204,205,206,207,208]. In the evolutionary context, robust traits enable degeneracy and drift to accumulate underneath, providing a ratchet which stabilizes the trait (since it is now required to tame the noise underneath). This could work similarly with memories: once there are degeneracies in the generalization/mapping of events->engram, the agent becomes complex to be able to actively interpret them, and can no longer easily go backwards—a ratchet for minds as well as for DNA/phenotypes.

48 Prediction-as-projection, and perceptual control theory, speak to this extensively [212,213,214]. This also has links to cell lineage competition in evolutionary developmental biology [215,216].

49 Paul Pietsch suggests that memory phase codes can be encoded in the resonance between atoms within living tissues [43]. James Shapiro [219] points out that there is no line of demarcation between informational and functional molecules in biology.

50 Note that unconventional memory media can include dynamical states, rather than physical substrates, as discussed in [220,221,222].

51 Computational simulations in the field of Artificial Life show the willingness of evolutionary processes to exploit all of the affordances of the environment [228,229].

52 A related model system would be general anesthesia—not only regarding cases of postoperative delirium [238,239], but also the broader question of how it is even possible for a mind to recover its state at all after the bioelectric network is interrupted and rebooted [240,241].

53 Perhaps small implants are sometimes able to override the patterns of a much larger body [247] because they work harder—being at risk of extinction, maybe they exert more effort to hack their host (their niche) to persist. There are data showing that cells which are in their correct positions with their anatomical setpoints satisfied complacently ignore global signals that are actively processed by cells that are in a precarious or uncertain state [125,126,248].

54 Moreover, the more robust, remappable, deep thoughts are the most agentially potent ones (it is not the physical agent that is robust; it is the behavioral, physiological, and anatomical memories that are). This view is the opposite of the superficially similar Dawkensian memes [249] because, I think, like in other aspects of biology, limiting oneself to a view in which propagating patterns are pawns of a purely mechanical process leads to many missed opportunities afforded by an agential lens [244,250].

55 The shelf which helps Huygens clocks achieve synchrony is a very basal example of a low-bandwidth hub node that helps active agents transfer information. This leads to a further question of whether wave patterns need a medium that is waving, or whether, like with electromagnetic waves and their back-and-forth action between the electric and magnetic components, it can be a self-reinforcing pattern that needs no “cogniferous aether” in the form of a brain in which to persist. While resonance is one way to understand the interaction of the elements of thought, Von Foerster suggests another, more geometric formalism for the chemistry of thought: tiling, in which “cognitive tiles” tesselate into larger patterns [82].

56 Niche construction is basically like stigmergy (memory), when we look at the evolutionary timescale. The actions and perceptions of an agent leave information in its environment, which then alters how it acts in the future—the environment is like a memory medium, external from the perspective of individual organisms but internal from the perspective of the lineage mind.

57 This is also a possible framework for understanding addictions to processes that remodel the mind to facilitate their own continuation. Another potential risk of systems that enable data to manipulate them is that this makes these systems susceptible to permanent changes [255].

58 It has already been proposed [259], in the case of an evolutionary-scale thinker, that the fundamental units of evolution are metabolic and developmental interaction *patterns*. A relevant science-fiction story concerns some extremely dense beings emerging from the center of the Earth, to whom we surface-dwellers are part of the gas phase—in this story, these beings cannot see us. One of them claims that he has been studying ripples in this gas that almost look like they have a degree of agency, despite the fact that they only persist for ~100 years and are easily disrupted by his swirling, but the others do not believe him as patterns in a gas cannot be a “thing”, much less an agential being, and certainly not ones that are formed and disappear in what to them is the blink of an eye. Thus, whether one sees patterns, or the physical medium in which they exist, as the agent, is dependent on the cognitive properties of that observer. Extending this idea, one might imagine alien beings wanting to communicate with life on Earth—would they try talking to people, or to our cells, or to our financial system, or to our ecosystems? Every cognitive being will have a preferred vantage point from which to recognize other minds, and it is essential to note that such vantage points are just evolutionary user interfaces [70,71,72], not privileged/correct ways to pick the right level of organization and cognition.

59 Perhaps the dichotomy between goals and goal-havers could be made into a continuum in the same way.

60 Perhaps all of the “stable objects” (including organisms) that we see are just the low-dimensional hub nodes—the medium bearing the pattern—and it is the persistent, deep pattern that is the true agent. This links to ideas we are developing on the active Platonic space concept, which focuses attention on the patterns and their interactions, not the media that they temporarily animate. On this view, we are just slightly coarser variants of our memories, all essentially denizens of Platonic space, projected temporarily into low-dimensional media.

61 Regarding how differently and for how long, that is a matter of degree, determined by (and determining) the system’s cognitive light cone—the spatiotemporal distance of events, from the here and now, that are needed to have a powerful understanding of a system.

62 Polycomputing emphasizes both the *read* side of the interaction (where agents perceive and interpret others’ activity in whichever way they want) and the *write* side of the interaction (where agents use this information to optimally direct the behavior of other systems in ways that bring adaptive benefits to them).
